# Feasibility study on intracranial pressure and prognosis of patients with moderate and severe craniocerebral injury using the Rotterdam computed tomography score: an observational study

**DOI:** 10.3389/fneur.2025.1554181

**Published:** 2025-03-26

**Authors:** Juan Ni, Wei Zhao, Zhifeng Wang, Xuejian Wang

**Affiliations:** ^1^Department of Nursing, Affiliated Hospital 2 of Nantong University, Nantong University, Nantong, Jiangsu, China; ^2^Department of Neurosurgery, Affiliated Hospital 2 of Nantong University, Nantong University, Nantong, Jiangsu, China

**Keywords:** craniocerebral injury, Rotterdam computed tomography score, coma, prognosis, appraised value, feasibility

## Abstract

**Objective:**

The Rotterdam computed tomography (CT) score was used to evaluate the degree of coma and the prognosis of patients with moderate and severe craniocerebral injury, to analyze its feasibility, and to assess its value in guiding further clinical applications.

**Methods:**

A total of 120 patients with moderate-to-severe craniocerebral injuries were selected as study participants, all of whom were treated at the Department of Neurosurgery of the Second Affiliated Hospital of Nantong University. All 120 patients underwent craniocerebral CT scans. The Glasgow Coma Scale was used to evaluate the degree of coma, and the Glasgow Outcome Scale was used to evaluate prognosis. The Rotterdam CT scores of patients with different degrees of coma and prognoses were compared.

**Results:**

The Rotterdam CT score was significantly lower in patients with moderate coma than in those with severe coma (*p* < 0.05). The Rotterdam CT score of patients with a good prognosis was significantly lower than that of patients with a poor prognosis (*p* < 0.05).

**Conclusion:**

The Rotterdam CT score is indicative of the degree of coma in patients with moderate and severe craniocerebral injuries and has prognostic value. The Rotterdam CT score also shows potential for broader clinical application.

## 1 Introduction

Traffic accidents and falls from buildings have increased with societal development. Craniocerebral injury is the most common cause of death and severe disability among various types of trauma. Patients with craniocerebral injuries often present in critical condition, with rapid changes, and have high mortality and disability rates. Therefore, early and accurate prognostic prediction is of great significance for clinical decision-making, treatment effect evaluation, and the reasonable allocation of medical resources (1–3). At present, the treatment of patients primarily relies on predicting the condition of patients with craniocerebral injury based on their Glasgow Coma Scale (GCS) score, which requires relevant physicians to evaluate the patients on-site. However, this method involves certain subjective factors that are vulnerable to external influences and lacks objectivity. It is therefore important to identify simple, intuitive methods to assess a patient’s condition. Computed tomography (CT), because of its convenience, speed, objectivity, and widespread availability, is widely used in clinical practice, including in primary hospitals. It is the first choice for diagnosing acute craniocerebral injuries. Currently, CT has become the main auxiliary diagnostic tool that informs further treatment. The selection of high-risk patients for intracranial pressure monitoring and early prognostic assessment based on CT scan results is of great significance in managing patients with craniocerebral injuries. It has been confirmed that some CT imaging features correlate with prognosis (4–7). Therefore, this study used the Rotterdam CT scoring system to further demonstrate the feasibility of this assessment method.

## 2 Data and methods

### 2.1 General data

Patients with moderate and severe craniocerebral injuries admitted to the Second Affiliated Hospital of Nantong University between February 2019 and October 2023 were selected as study participants. This study was approved by the Medical Ethics Committee of our hospital.

The inclusion criteria were as follows: age > 16 years and < 75 years; a clear history of brain trauma; acute moderate-to-severe craniocerebral injury (GCS score of 3–12 points); time from injury to visiting our hospital <12 h; acceptable follow-up; and informed consent signed by the guardian.

Exclusion criteria included serious heart, lung, liver, kidney, or other chronic diseases (including heart failure, emphysema, hepatitis, kidney failure, and uremia); coexisting neurological diseases (including Alzheimer’s disease); hematological diseases (including hemophilia); intracranial tumors; a history of stroke; serious injuries to other organs; pregnancy or lactation; and withdrawal of treatment because of hospital transfer or other reasons.

Of the 120 patients initially planned for inclusion in this study, 17 were later excluded for various reasons (including severe infections and hospital transfers). A total of 103 cases were included in the analysis. There were 76 men and 27 women. The average age was 46.31 ± 10.24 years, ranging from 18 to 72 years. The time from injury to admission ranged from 30 min to 9 h, with an average of 2.13 ± 1.32 h. Causes of injury included: 52 cases of traffic accidents, 21 cases of falls from high places, 13 cases of fall-related trauma, 10 cases of heavy crushing injuries, and seven cases of violent blows. Of these, 78 patients underwent surgical treatment.

### 2.2 Methods

All 103 patients with moderate and severe craniocerebral injuries underwent craniocerebral CT scans using a 256-slice spiral CT scanning machine (GE Company, USA). The scanning time was 2–3 s, and the scanning plane was parallel to the canthomeatal line, with one layer every 5 mm. The Rotterdam CT scoring method was used by two experienced physicians to evaluate the images based on midline shift, basal cisterna status, bleeding status, and other signs.

### 2.3 Research indicators

**Figure 1:**
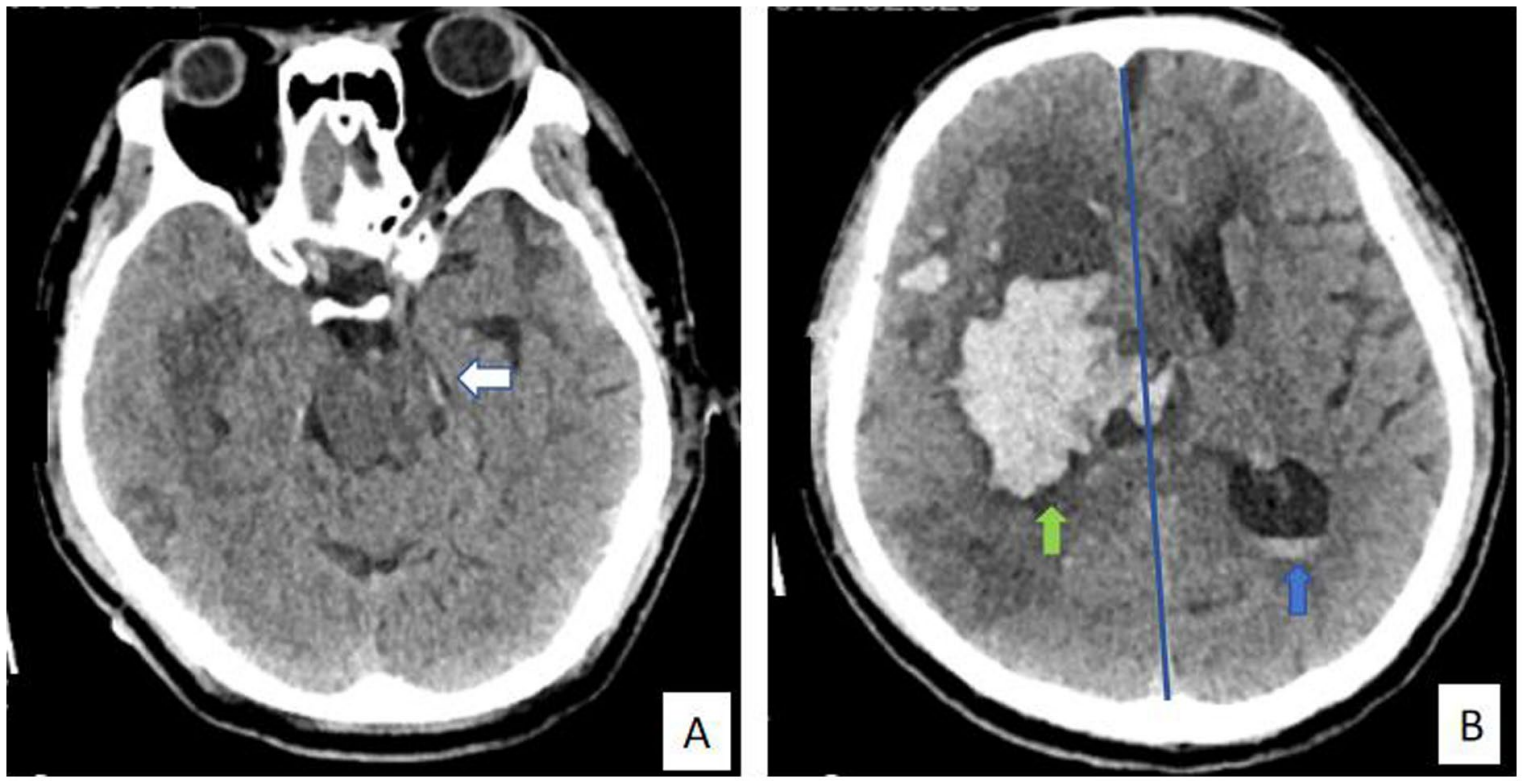
**(A, B)** A 66-year-old male patient with cerebral hemorrhage was admitted to the hospital and underwent surgical treatment. The final prognosis was not good. Rotterdam CT score are as follows: basal cisterna status: one point for compression (white arrow); midline displacement: one point for a shift >5 mm (blue line); ventricular hemorrhage: one point for presence (blue arrow); and space-occupying lesions, such as hematomas or contusions: one point for presence (green arrow). The total score plus one resulted in five points, which was used to predict prognosis.

**Figure 2:**
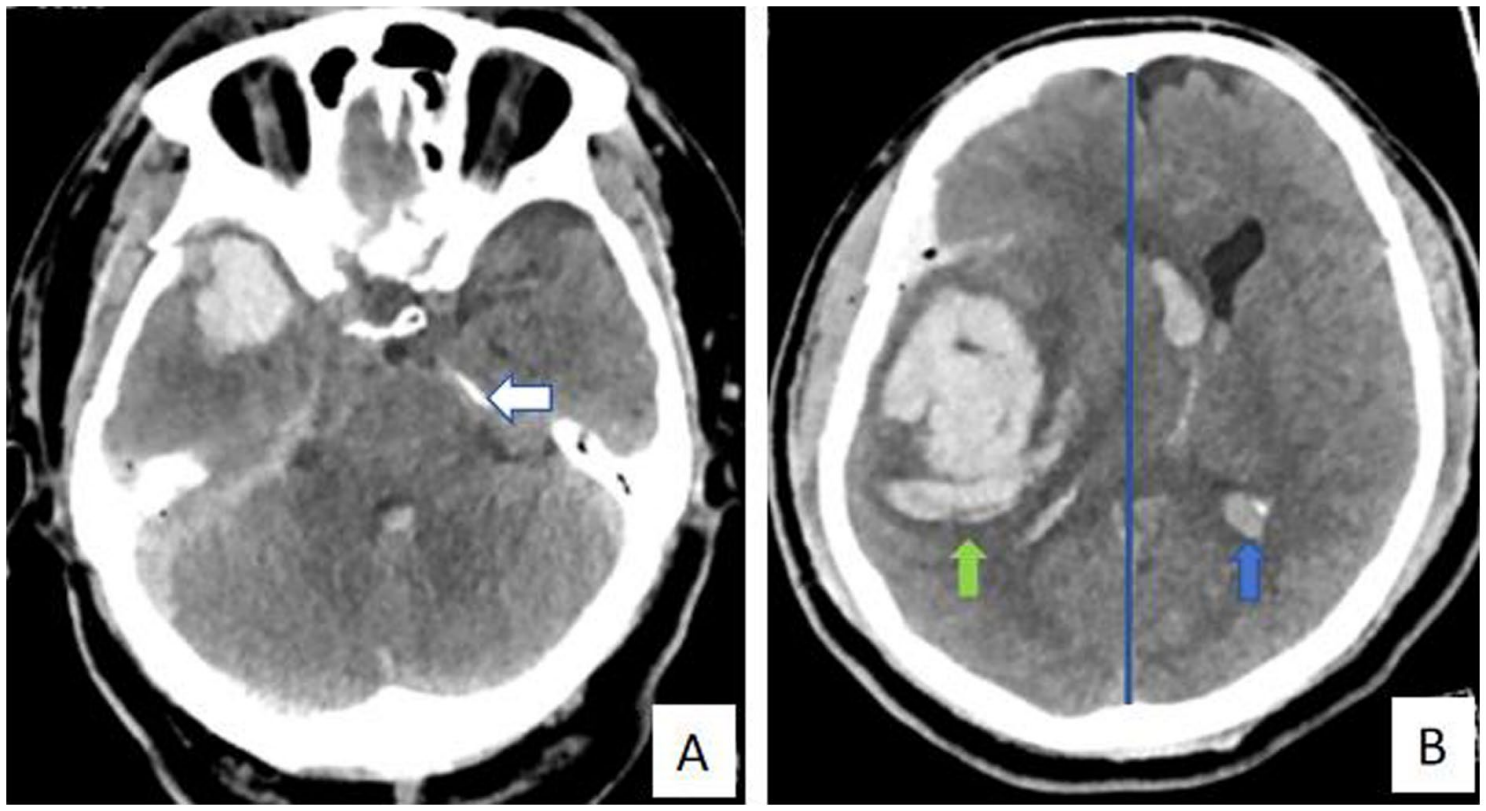
**(A, B)** A 60-year-old male patient with brain trauma was admitted to the hospital and underwent surgical treatment. The final prognosis was not good. Rotterdam CT score are as follows: basal cisterna status: two points for displacement (white arrow); midline displacement: one point for a shift >5 mm (blue line); ventricular hemorrhage or traumatic subarachnoid hemorrhage: one point for presence (blue arrow); and space-occupying lesions, such as hematomas or contusions: one point for presence (green arrow). The total score plus one resulted in six points, which was used to predict prognosis.

(1) Rotterdam CT score: The score was determined based on CT findings. Scoring criteria were as follows: basal cisterna status: two points for displacement, one point for compression, and zero points for normalcy; midline displacement: one point for a shift >5 mm and zero points for a shift ≤5 mm; ventricular hemorrhage or traumatic subarachnoid hemorrhage: one point for presence and zero points for absence; and space-occupying lesions, such as hematomas or contusions: one point for presence and zero points for absence. The total score plus one resulted in a maximum of six points, which was used to predict prognosis (8, 9) (Typical cases: Figures 1A,B, 2A,B). (2) Coma degree: The degree of coma was assessed using the GCS. The scale includes 15 items across the dimensions of speech, movement, and eye-opening, with a score range of three to 15 points. Scores ≥13, 9–12, and 3–8 were classified as mild, moderate, and severe comas, respectively (10). (3) Prognosis: Patients were followed up for 6 months after treatment using the Glasgow Outcome Scale (GOS). Prognosis was classified as follows: death, plant survival, severe disability (inability to live independently, requiring care), moderate disability (ability to live independently but requiring protective work conditions), and good recovery (ability to live independently with normal work and life). Scores 4–5 were classified as a good prognosis, whereas scores 1–3 were classified as a poor prognosis (11).

### 2.4 Statistical analysis

SPSS 26.0 statistical software was used for analysis. Statistical data were expressed as rates (%). Measurement data conforming to a normal distribution were expressed as mean ± standard deviation (x ± s), and a *T*-test was used for comparison. Statistical significance was set at *p* < 0.05.

## 3 Results

### 3.1 Comparison of surgery requirements with Rotterdam CT scores

Among the 103 patients with moderate-to-severe craniocerebral injury, 78 (75.73%) underwent surgery and 25 (24.27%) did not. The Rotterdam CT scores of surgical patients were compared with those of nonsurgical patients, and the difference was statistically significant (*p* < 0.05) (Table 1).

**Table 1:** Comparison of research indexes and Rotterdam CT scores (x ± s, points).

Index	Group	Num	Rotterdam CT score	*t*-value	*p*-value
Repuiers surgery or no	Surgery	78	4.96 ± 0.71	12.489	<0.05
	No Surgery	25	3.01 ± 0.57		
Degree of coma	Middle coma	36	3.34 ± 0.75	9.448	<0.05
	Severe coma	67	4.83 ± 0.77		
Prognosis	Favorable prognosis	71	3.24 ± 0.68	9.297	<0.05
	Poor prognosis	32	4.67 ± 0.81		

### 3.2 Comparison of Rotterdam CT scores in patients with different degrees of coma

Among the 103 patients with moderate-to-severe craniocerebral injury, 36 (34.95%) were in a moderate coma and 67 (65.05%) were in a severe coma. The Rotterdam CT scores of patients in moderate coma were compared with those of patients in severe coma, and the difference was statistically significant (*p* < 0.05) (Table 1).

### 3.3 Comparison of Rotterdam CT scores in patients with different prognoses

Among the 103 patients with moderate-to-severe craniocerebral injury, 71 (68.93%) had a good prognosis, and 32 (31.07%) had a poor prognosis. The Rotterdam CT scores of patients with a good prognosis were compared with those of patients with a poor prognosis, and the difference was statistically significant (*p* < 0.05) (Table 1).

## 4 Discussion

Craniocerebral injury is a common condition in neurosurgery with a high disability and fatality rate. It seriously affects the survival and prognosis of patients and garners substantial clinical attention (12). Despite improvements in living standards and the rapid development of mechanical engineering, transportation, construction, and other industries, its incidence remains high. As the conditions of patients with craniocerebral injury are complex and changeable, timely and accurate diagnosis and evaluation are essential prerequisites for ensuring effective treatment (13). CT is the main examination method for craniocerebral injury because it can visually display the correlation between patients’ intracranial tissues (14). Rotterdam CT scoring is a new CT classification method proposed by Maas et al. (15). Maas randomly evaluated the injury condition and prognosis of patients with craniocerebral injury and found that this classification method was more effective than the Marshall CT classification method proposed earlier (14, 16). The Rotterdam CT score quantifies multiple CT signs, such as midline displacement, intracranial hemorrhage, and basal cisternal compression. It has the advantages of convenient calculation, good repeatability, and high specificity and sensitivity (17, 18). There are some limitations in evaluating injuries based on the characteristics of individual CT images. For example, in cases of diffuse craniocerebral injury with diffuse swelling of brain tissue, the midline may not be displaced or may be slightly displaced even when the injury is severe and intracranial pressure is high, resulting in inaccurate predictions. The Rotterdam CT score synthesizes all the important features of CT images, enabling it to reflect the real situation of the injury (19–21).

In this study, 78 of 103 patients with moderate and severe craniocerebral injury required surgical treatment, accounting for 75.73%. For these patients, the primary consideration is to save lives after surgery, but this also indicates that their condition is relatively severe. The Rotterdam CT scores of surgical patients were compared with those of non-surgical patients, and the difference was statistically significant (*p* < 0.05). This finding demonstrates that patients with high Rotterdam CT scores have more severe disease and a high risk of requiring surgery (17, 22). Patients with moderate and severe craniocerebral injuries tend to exhibit more pronounced brain tissue swelling and hematomas, resulting in increased intracranial pressure and compression of the midline structure, basal cisterna, and ventricles (18, 21, 23). Therefore, higher Rotterdam CT scores correlate with more severe disease.

The GCS has long been recognized as an important tool for evaluating the degree and severity of coma in patients with craniocerebral injury and is widely used clinically (10). In this study, among the 103 patients with moderate and severe craniocerebral injuries, 34.95% were in moderate coma (GCS score of 9–12 points), and 65.05% were in severe coma (GCS score of 3–8 points). The Rotterdam CT scores of patients in severe coma were significantly higher than those of patients in moderate coma. The difference was statistically significant (*p* < 0.05). These results indicate that the Rotterdam CT score objectively reflects the degree of coma in patients with moderate and severe craniocerebral injuries and provides a reference for diagnosing the condition.

Reducing adverse prognosis and improving the quality of life of patients are the main goals of clinical treatment for craniocerebral injuries. The Rotterdam CT score can serve as an independent predictor of adverse prognosis in patients with craniocerebral injury (24, 25). In this study, after a 6-month follow-up of patients with moderate and severe craniocerebral injury, it was found that among the 103 patients, 68.93% had a good prognosis (GOS score 4–5 points), and 31.07% had a poor prognosis (GOS score 1–3 points). The Rotterdam CT scores of patients with a poor prognosis were significantly higher than those of patients with a good prognosis (*p* < 0.05). These results indicate that the Rotterdam CT score has predictive value for the prognosis of patients with craniocerebral injuries. This may be attributed to the severity of craniocerebral injury and brainstem involvement, which lead to limb motor function impairment and consciousness disturbances, ultimately affecting prognosis.

In summary, the Rotterdam CT score is closely related to whether patients with moderate or severe craniocerebral injury require surgery, their degree of coma, and their GOS score. This scoring system provides valuable guidance for assessing patient condition and prognosis.

## Data Availability

The original contributions presented in the study are included in the article/supplementary material, further inquiries can be directed to the corresponding author.
